# Nanoscale Polishing of TC4 Titanium Alloy Surface Based on Dual-Pole Magnetic Abrasive Finishing Method

**DOI:** 10.3390/mi16060620

**Published:** 2025-05-25

**Authors:** Zhenfeng Zhou, Xu Sun, Shibing Liang, Ying Fang, Yanzhen Yang, Yongjian Fu, Shiqing Zou

**Affiliations:** 1 College of Information Science and Engineering, Jiaxing University, Jiaxing 314001, China; zzf@zjxu.edu.cn; 2 College of Physics and Mechanical Engineering, Longyan University, Longyan 364000, China; 13850618500@163.com (S.L.); fy@lyun.edu.cn (Y.F.); yyz@lyun.edu.cn (Y.Y.); fyj@lyun.edu.cn (Y.F.); 3 Xiamen Tungsten Intelligent Equipment Company, Xiamen 361000, China; zou.shiqing@cxtc.com

**Keywords:** dual-pole magnetic abrasive finishing, TC4 titanium alloy, surface finishing, machining parameters, surface roughness

## Abstract

The dual-pole magnetic abrasive finishing (DMAF) method was proposed to achieve a smooth surface on TC4 titanium alloy. Firstly, both the distribution of the magnetic field and the intensity of magnetic induction produced by nine combinations of magnetic poles of different shapes were simulated using Ansys Maxwell software (2024R2). According to the results of the simulation, the optimal combination of magnetic poles was determined. Then, the machining parameters of multi-stage DMAF were optimized through comparative experiments on major single factors. Finally, combinations of the mixed magnetic abrasive in three polishing stages were obtained as follows: #100 electrolytic iron powder (Fe_3_O_4_) + #2000 white abrasive (WA), #200 Fe_3_O_4_ + #8000 WA, and #450Fe_3_O_4_ + #w1 diamond (DMD). The gap between the upper and lower magnetic poles was set to 5 mm, the rotational speed of the magnetic pole was set to 300 rpm, and the quality ratio of the abrasive was 2:1. The experiments indicated that the average surface roughness Ra was reduced from an initial value of 0.433 μm to 8 nm after 30 min of multi-stage DMAF, and a nano-level mirror polishing effect was essentially achieved in the polishing zone.

## 1. Introduction

TC4 titanium alloy, as an “α + β” type of titanium alloy, is widely used in many industries, such as aerospace, biomedicine, ocean engineering, the automobile industry and engineering, etc., owing to its characteristics of a low density, high temperature resistance, excellent corrosion resistance, and superior vibration damping capabilities. For example, TC4 titanium alloy is mainly used to make complex parts such as aircraft engines, airframes, turbine blades, and integral impellers in the aerospace field [1,2]. The surface roughness of such parts will affect the surface quality and reliability of an aircraft engine’s components, the fatigue strength of the parts, the ability to suppress vibration and noise, the performance of fuel supply systems, and the stability of hydraulic systems [3,4]. However, due to the high strength, ductility, and low thermal conductivity of the TC4 titanium alloy, traditional mechanical processing methods, such as sand belt grinding and milling, are prone to causing the workpiece surface to harden. Moreover, an increasing processing temperature can scorch the workpiece surface, resulting in an increase in its surface roughness and processing texture, which will have adverse effects on the fitting accuracy and fatigue strength of the part [5,6]. Evidently, precision machining of TC4 titanium alloy material has remained a huge global challenge.

Magnetic abrasive finishing (MAF), a special precision machining method, harnesses the energy of a magnetic field to propel magnetic abrasive media (such as stainless steel needles, electrolytic iron powder, etc.) to collide and rub against a workpiece’s surface at high speed, enabling efficient deburring and surface polishing. In the 1980s, Shinmura et al. were the first to conduct comprehensive research into and put into practice the polishing principles, device development, the polishing process, and parameter experiments for MAF [7,8,9,10]. Their extensive experimental results demonstrated the MAF process as an effective precision machining method. However, the mixed magnetic abrasive during the MAF process in a free slurry state leads to inadequate rigidity and a much lower polishing efficiency compared to that in traditional mechanical machining [11,12,13]. To address the unevenness of the polishing pressure, some researchers have not only improved the magnetic machining tools and optimized the polishing parameters but have also proposed composite machining methods combining the MAF process with other energy fields. Fang et al. established mathematical models of the distribution of the magnetic field in MAF and analyzed the dynamic behavior of the magnetic abrasive under a magnetic field. They also simulated the characteristics of the distribution of the magnetic field for grooved poles and explored the single-particle distribution law of the magnetic force, the interaction force, and interface pressure [14]. Yamaguchi et al. suggested a multiple-pole-tip system operating at high rotational speeds (up to 30,000 min^−1^) for internal polishing of capillary tubes, demonstrating significant efficiency improvements [15,16]. Zou et al. developed an auxiliary magnetic jig for polishing the inner surfaces of welded, thick-walled tubes. Their experimental results revealed that the surface quality was obtained in a shorter polishing time because the combined external and internal magnetic fields enhanced the machining force, achieving a superior surface quality in a reduced time [17,18]. Xie et al. utilized an alternating magnetic field with full-wave rectification to enhance the polishing efficiency of the MAF process, demonstrating that dynamic reorientation of the free abrasive particles improved their material removal capacity [19]. Sun et al. proposed a compound polishing method, electrochemical magnetic abrasive finishing (EMAF), for polishing SUS304 stainless steel [20,21,22]. Under the electrochemically assisted effect, a passive film formed on the surface of the metal workpiece. Because the hardness of the passive film was much lower than that of the metal surface, the material removal capacity of the abrasive particles was significantly improved. This resulted in a 75% improvement in the polishing efficiency compared to that of traditional MAF. Moreover, the hybrid process of ultrasonic-assisted magnetic abrasive finishing (UMAF) has been studied for polishing hard metal or alloy materials by some researchers [23,24]. Their experimental results revealed that the hybrid process of UMAF shortened the processing time while reducing the surface roughness to the extreme. These studies have collectively improved the polishing efficiency of the MAF process through diverse approaches.

To improve the polishing pressure, a dual-magnetic-pole magnetic finishing (DMAF) method was proposed for precision surface finishing of the TC4 titanium alloy. Firstly, nine different combinations of magnetic poles were designed. Ansys Maxwell finite element software was used to simulate the distribution of the magnetic field and the intensity of the magnetic induction generated by nine combinations, and the optimal combination of magnetic poles was determined through a comparison. By conducting comparative experiments on the main single factors, such as the abrasive combination, the working gap between magnetic poles, and the rotational speed of the magnetic poles, the processing parameters of multi-stage DMAF were optimized. Subsequent multi-stage dual-pole magnetic abrasive finishing experiments were conducted on TC4 titanium workpieces.

## 2. Methods and Experimental Procedures

### 2.1. Analysis of Magnetic Field Strength

#### 2.1.1. The Shape and Combination of the Magnetic Poles

**Figure 1:**
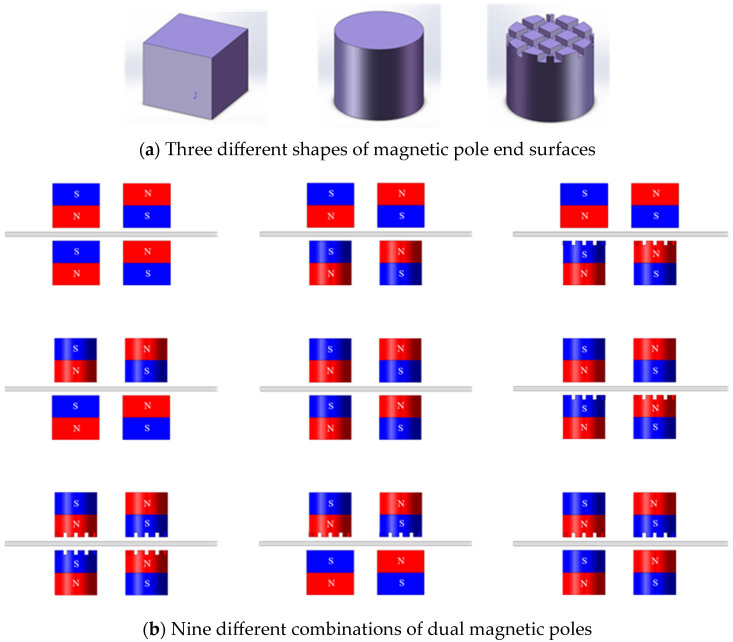
Shapes and combinations of dual magnetic poles.

The shape of a magnetic pole’s end face significantly influences the distribution of its spatial magnetic field, which in turn determines the surface quality and polishing efficiency of MAF [25,26,27]. Therefore, this study focuses on the shapes and combinations of the end faces of magnetic poles. Figure 1 shows nine different combined dual magnetic poles with three different end face shapes. The permanent magnetic poles are designed as cube, cylinder, and 90°-slotted cylinder shapes. The cubic permanent magnetic pole has a side length of 10 mm. The cylindrical permanent magnetic pole has a circular cross-section with a diameter of 9 mm and a height of 10 mm. The 90°-slotted cylindrical permanent magnetic pole has a circular cross-section with a diameter of 9 mm, a height of 10 mm, a groove depth of 0.8 mm, and a groove pitch of 1 mm.

#### 2.1.2. Simulation Analysis of the Magnetic Pole Combinations

**Figure 2:**
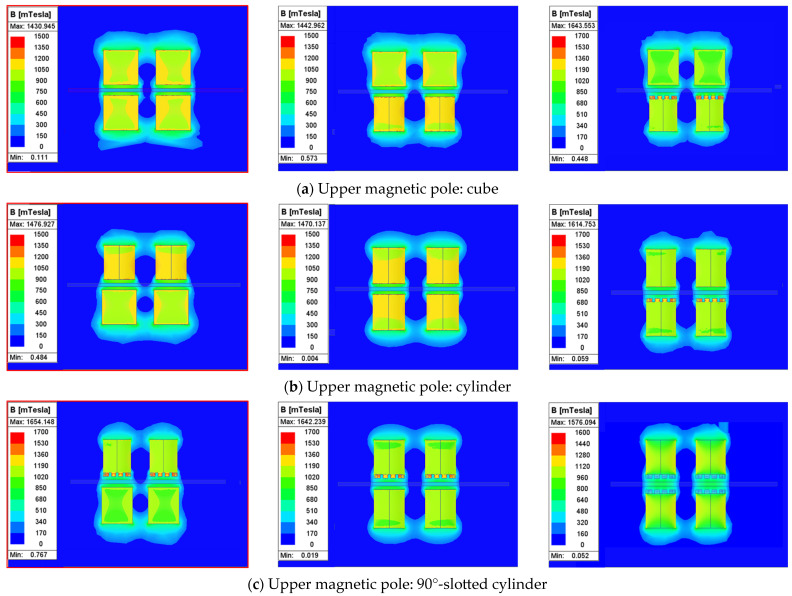
The magnetic field distribution and magnetic induction intensity of nine different combinations of dual magnetic poles. (**a**) Magnetic field distribution and magnetic induction intensity between a cubic upper magnetic pole and a cubic lower magnetic pole; (**b**) magnetic field distribution and magnetic induction intensity between a cylindrical upper magnetic pole and a cubic lower magnetic pole; (**c**) magnetic field distribution and magnetic induction intensity between a 90°-slotted cylindrical upper magnetic pole and a cubic lower magnetic pole.

In order to quantitatively analyze the distribution of the magnetic field and the intensity of magnetic induction with the nine types of dual-pole magnetic machining tools, we used Ansys Maxwell simulation software to establish cloud maps of the distribution of the magnetic fields and determine the magnetic induction intensities of different magnetic pole combinations. A natural boundary with a boundary value of 200 was selected as the boundary condition, and the mesh was discretized with an element size of 1 mm. The upper and lower magnetic poles were made of NdFe35, and the workpiece was set as TC4 titanium alloy. The results of the simulation analysis are presented in Figure 2.

Among the three dual magnetic pole combinations with the cubic upper magnetic pole shown in Figure 2a, the maximum 0.792 T magnetic induction intensity in the processing zone occurred when the cubic upper magnetic pole was paired with a lower cubic pole. Among the three dual magnetic pole combinations with the upper cylindrical magnetic pole shown in Figure 2b, the maximum 0.779 T magnetic induction intensity in the processing zone occurred when the upper cylindrical magnetic pole was paired with a lower cubic magnetic pole. Among the three dual magnetic pole combinations with the 90°-slotted cylindrical upper magnetic pole shown in Figure 2c, the maximum 0.701 T magnetic induction intensity in the processing zone occurred when the 90°-slotted cylindrical upper magnetic pole was paired with a lower cubic magnetic pole. Collectively, these results demonstrate that the combination of upper–lower cubic magnetic poles maximizes the magnetic induction intensity in the processing zone. In addition, the simulation results indicate that the “N-S-N-S” closed magnetic field loop formed by the dual magnetic pole combination enhances the magnetic induction intensity in the processing zone.

### 2.2. The Machining Principle

**Figure 3:**
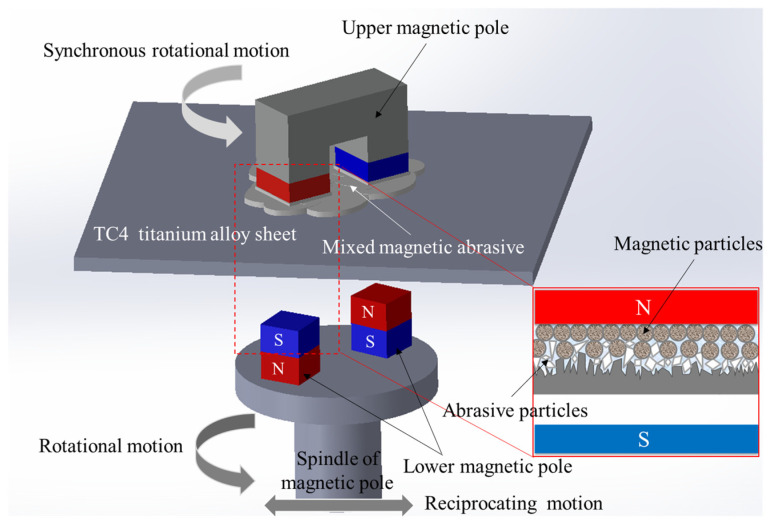
Machining principle of DMAF.

The machining principle of DMAF is shown in Figure 3. The magnetic machining tool comprises an upper magnetic pole pair featuring opposing polarities on the lower end faces and a lower magnetic pole pair exhibiting reversed polarities on the upper end faces relative to the upper magnetic pole. Thus, an “N-S-N-S” closed magnetic field loop polarity is formed in the polishing zone. During the process, magnetic abrasive particles align along the magnetic flux lines under the action of the force of the magnetic field, forming a “magnetic brush” on the end face of the magnetic pole. The upper magnetic pole synchronized the rotation with the lower magnetic pole and the axial reciprocating motion along the ball screw. Nanoscale precision polishing is attained through the controlled interfacial friction between the magnetic abrasive particles and the workpiece surface.

### 2.3. The Experimental Setup

**Figure 4:**
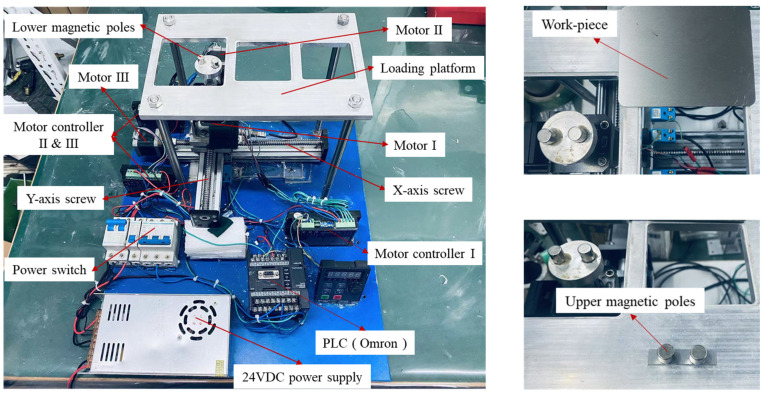
Experimental setup of DMAF plate.

Figure 4 shows the DMAF experimental setup, which integrates the following key components: three stepper motors, three motor drivers, an Omron PLC controller, ball screw assemblies, a 24 V DC power supply, a load platform, a pair of magnetic poles, a displacement restrictor, and a power switch. The load tray, rigidly coupled to motor I via helical beam coupling, houses the lower magnetic pole, whose kinematic trajectory is governed by the PLC controller. Stepper motors II and III, respectively, drive the ball screw assemblies for reciprocal linear motion along the X- and Y-axes. The work platform is designed with three different sizes to accommodate plate workpieces of varying dimensions. The TC4 titanium plate is placed on the work platform. A certain proportion of the mixed magnetic abrasive is evenly applied to the bottom of the upper magnetic pole, and the upper magnetic pole is placed on the workpiece. When the power is turned on, the rotational speed of the magnetic pole is regulated via the controller, with the lower magnetic pole inducing synchronous rotation of the upper magnetic pole through magnetic coupling. The mixed magnetic abrasive between the upper magnetic pole and the workpiece creates relative friction on the workpiece surface, thereby achieving precision machining of the workpiece surface. The abrasive needs to be replaced at 10 min intervals during polishing cycles to maintain the polishing performance.

### 2.4. The Experimental Conditions and the Measuring Method

The polishing object in this study is a TC4 titanium alloy plate specimen with a length of 100 mm, a width of 80 mm, and a thickness of 1 mm. The initial surface roughness Ra is approximately between 0.4 and 0.5 μm. In the single-factor comparative experiment, we mainly focused on the effects of the abrasive combination, the gap between the upper and lower magnetic poles, and the rotational speed of the magnetic pole on the polishing effect on the workpiece surface. The specific machining parameters are shown in Table 1. The mixed magnetic abrasive is made by mixing the iron-based phase (Fe_3_O_4_ electrolytic iron powder) with the polishing phase (Al_2_O_3_ white abrasive) at a mass ratio of 2:1 using a quantitative diamond oil-based polishing solution.

**Figure 5:**
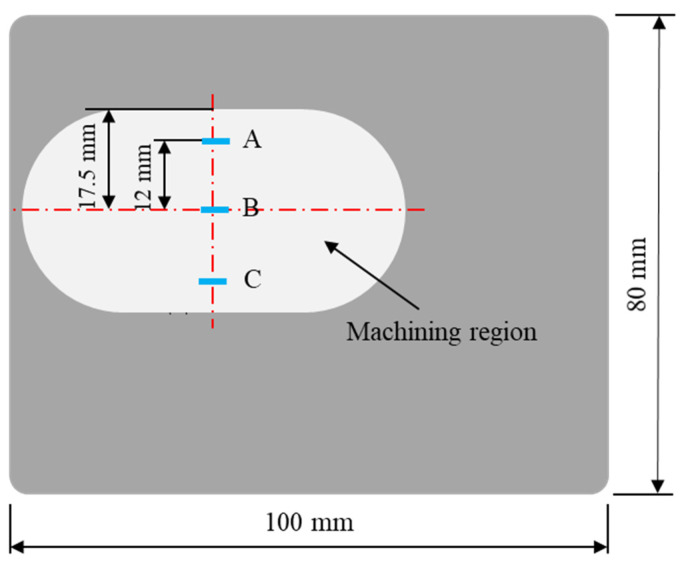
Measurement points on surface of TC4 workpiece.

After each polishing stage, the workpiece was cleaned using an ultrasonic cleaning machine to completely remove residual surface abrasive and chips from the workpiece. Then, the surface roughness Ra of the workpiece was measured using a contact-surface-roughness-measuring instrument (SHT-80, Fangyuan, Suzhou, China). During the final polishing stage, besides measuring the surface roughness Ra, the 3D morphology of the workpiece surface also needed to be observed using a white light interferometer (VT-X100, CHOTEST, Shenzhen, China). The surface roughness Ra and the 3D surface morphology were, respectively, measured and observed at three measurement points (A: upper; B: middle; C: lower) along the axis in the polishing zone, as shown in Figure 5.

## 3. Results

### 3.1. The Influence of the Gap Between the Upper and Lower Magnetic Poles on the Polishing Effect

**Figure 6:**
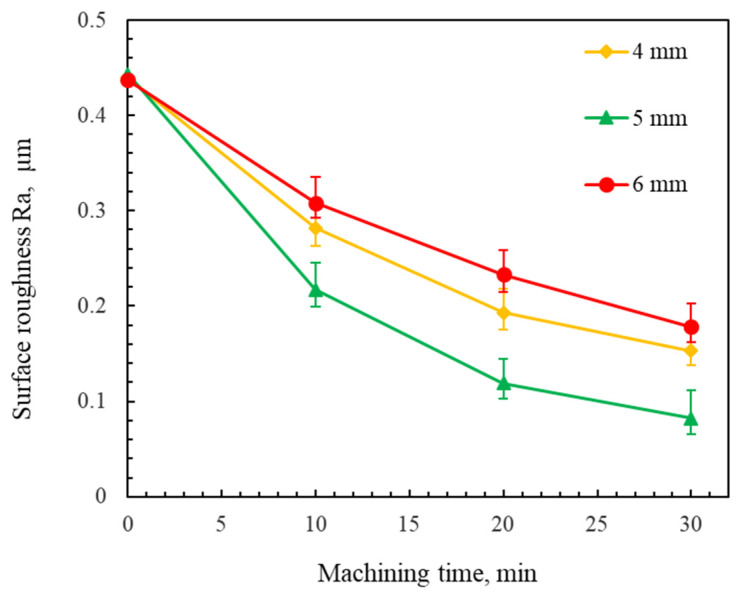
Changes in the surface roughness of the workpiece using different gaps between the upper and lower magnetic poles (using #100Fe_3_O_4_ + #2000WA mixed magnetic abrasive at a 200 rpm rotational speed).

Figure 6 shows the changes in the surface roughness of the workpiece using different gaps between the upper magnetic pole and the lower magnetic pole. The experimental results show that the surface roughness Ra achieved with a 5 mm gap between the magnetic poles is lower than that obtained with 4 mm and 6 mm gaps between the magnetic poles. As the gap between the upper magnetic pole and the lower magnetic pole increases, the strength of the magnetic field gradually decreases, resulting in a decrease in the polishing pressure of the abrasive particles. During polishing, a higher polishing pressure generally increases the material removal rate. However, an excessive polishing pressure induced scratch defects on the workpiece surface. Moreover, an insufficient polishing pressure reduced the material removal rate. These two situations resulted in an increase in the surface roughness of the workpiece. Thus, an optimal surface quality was achieved at a 5 mm gap between the upper magnetic pole and the lower magnetic pole.

### 3.2. The Influence of the Rotational Speed of the Magnetic Poles on the Polishing Effect

**Figure 7:**
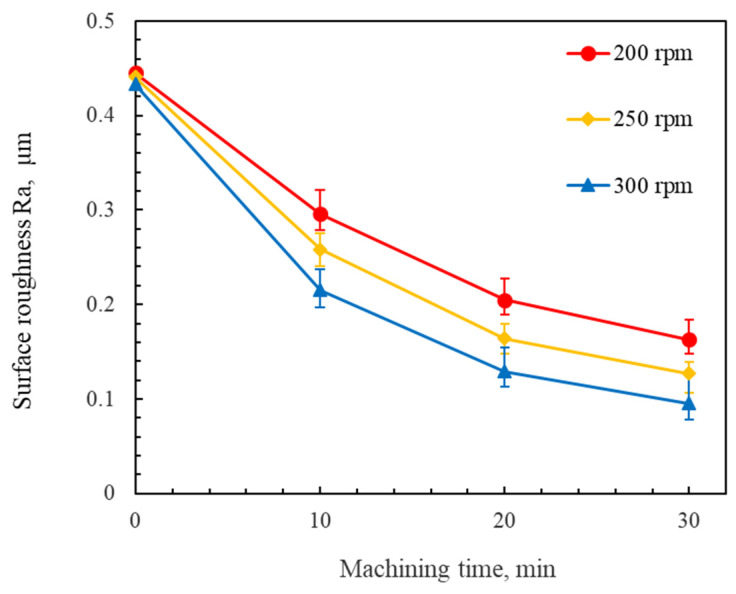
Changes in the surface roughness of the workpiece at different rotational speeds of the magnetic poles (using #100Fe_3_O_4_ + #2000WA mixed magnetic abrasive with a 6 mm machining gap).

Figure 7 shows the changes in the surface roughness of the workpiece at different rotational speeds of the magnetic poles. The experimental results show the rate of the decline in the surface roughness at a 300 rpm rotational speed of the magnetic pole is significantly higher than that at the other rotational speeds. As the rotational speed of the magnetic pole increases, the surface roughness Ra of the workpiece obtained decreases. It was also found that dynamic instability in the upper magnetic pole occurred when the rotational speeds of the magnetic poles exceeded 300 rpm. If the rotational speed of the magnetic pole spindle is too high, the free abrasive particles tend to detach from the magnetic field under the action of the centrifugal force and even splash out. The number of abrasive particles involved in polishing decreased as the rotational speed of the magnetic pole increased, which resulted in a decrease in the polishing efficiency. Hence, the 300 rpm condition provided the optimal process stability and efficiency.

### 3.3. The Influence of the Abrasive Combination on the Polishing Effect

**Figure 8:**
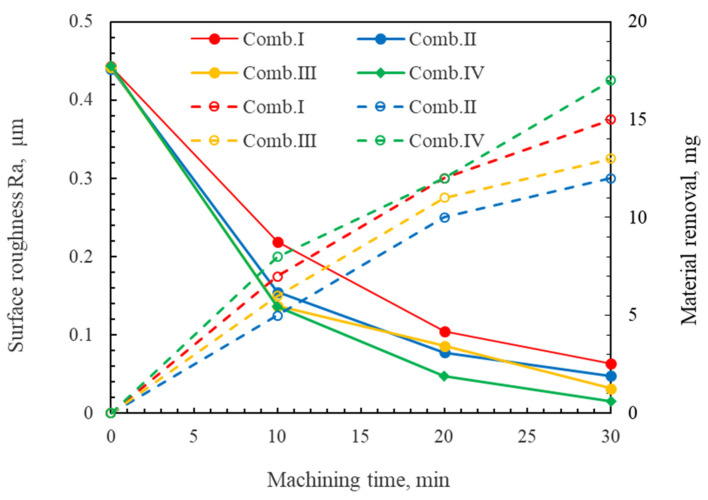
Changes in the average surface roughness and material removal amount for workpieces under different abrasive combinations.

During the MAF process, the size of the abrasive determines the surface roughness of the workpiece after each polishing stage [28]. Building on this foundation, we also evaluated the material removal amount using four different abrasive combinations (i.e., **Combination I:** #45Fe_3_O_4_ + #800WA, #100Fe_3_O_4_ + #2000WA, #200Fe_3_O_4_ + #4000WA; **Combination II:** #45Fe_3_O_4_ + #2000WA, #100Fe_3_O_4_ + #4000WA, #200Fe_3_O_4_ + #8000WA; **Combination III:** #100Fe_3_O_4_ + #2000WA#, 200Fe_3_O_4_ + #4000WA, #450Fe_3_O_4_ + #W2DMD; and **Combination IV:** #100Fe_3_O_4_ + #2000WA, #200Fe_3_O_4_ + #8000WA, #450Fe_3_O_4_ + #W1DMD) through multi-stage DMAF experiments. The changes in the surface roughness and material removal amount after DMAF polishing are shown in Figure 8.

The experimental results show that using abrasive combination IV achieved the minimum average surface roughness Ra of 0.015 μm of the workpiece, representing a reduction from the initial 0.433 μm. In the first polishing stage, the #800WA abrasive demonstrated a substantial amount of material removal but a limited reduction in the surface roughness. However, transitioning to the #2000WA abrasive reduced the surface roughness Ra to below 0.15 μm. In the second polishing stage, the #8000WA abrasive achieved a lower surface roughness Ra. In the final polishing stage, the surface roughness Ra obtained by using diamond abrasive with a smaller size (#15,000) was less than 20 nm since the hardness of the diamond abrasive was higher than that of the white abrasive. Additionally, it was also revealed that the size of the iron powder particles had little effect on the surface roughness of the workpiece compared to the size of the abrasive particles used during the three polishing stages.

### 3.4. Characterization of the Workpiece Surface After Multi-Stage Polishing

**Figure 9:**
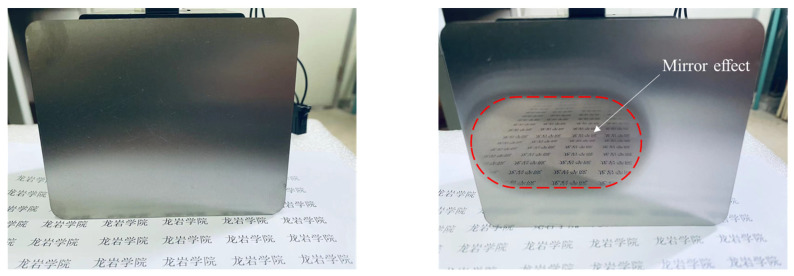
Physical images of TC4 titanium alloy workpiece before and after polishing. (龙岩学院means Longyan University).

Physical images of the TC4 titanium alloy workpieces before and after polishing are shown in Figure 9. The comparative results show that a mirror-like polishing effect in the polishing zone was basically achieved after 30 min using the multi-stage DMAF method.

**Figure 10:**
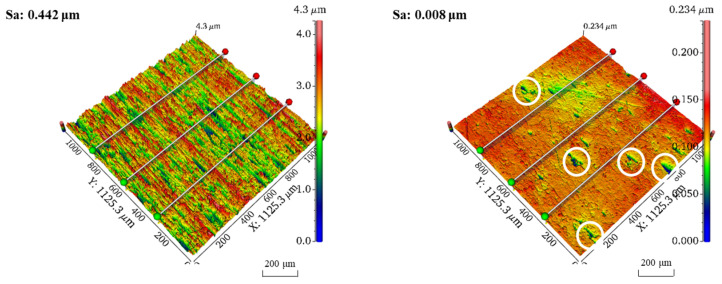
Comparison of three-dimensional morphology at measurement point B of workpiece before and after polishing.

Finally, the surface morphology of the TC4 titanium alloy workpieces was evaluated using a white light interferometer. Results on the 3D morphology of point B before and after polishing are shown in Figure 10. The average surface roughness Sa of point B decreased from an initial value of 0.442 μm to 8 nm after 30 min of polishing. The comparative results reveal that the 3D morphology of point B after polishing basically presented a flattening trend, but there were still a small number of defects (with a max size of less than 80 μm), marked with white circles on the workpiece surface. The peak-to-valley height at point B decreased from 4.3 μm before polishing to 0.234 μm after polishing.

### 3.5. Simulation of the Intensity of Magnetic Induction 

**Figure 11:**
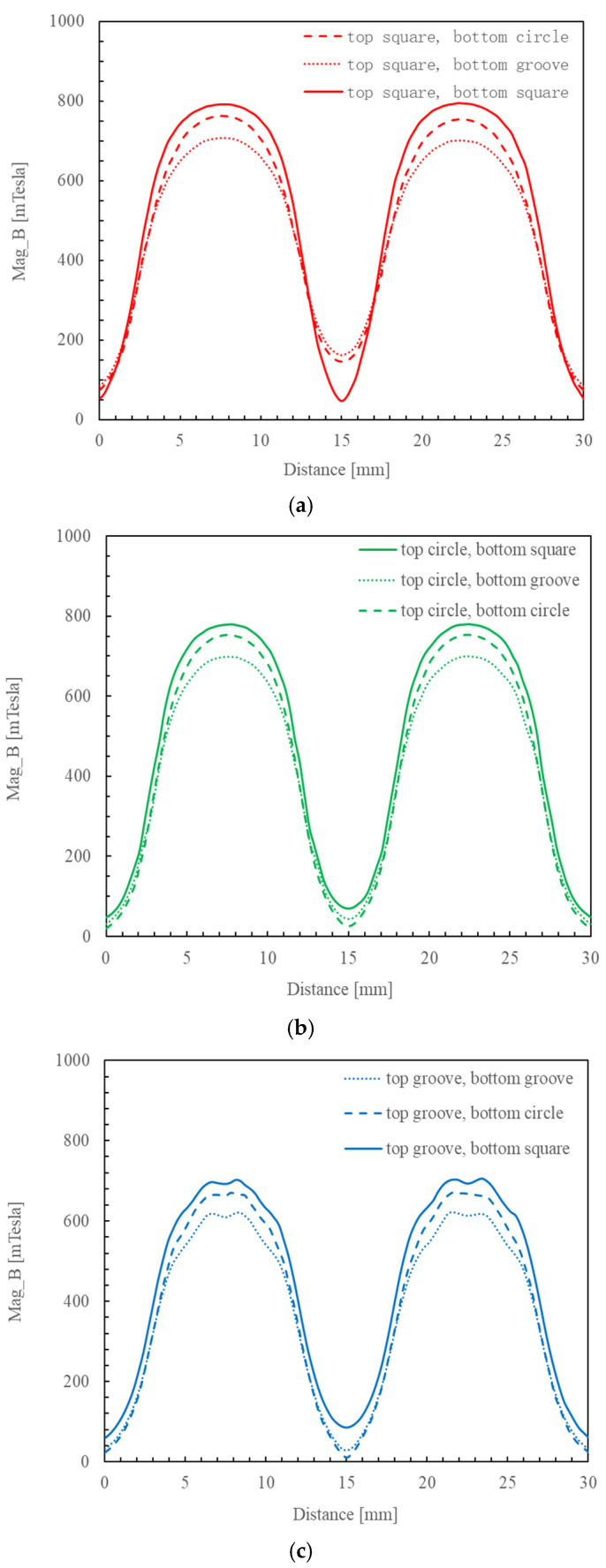

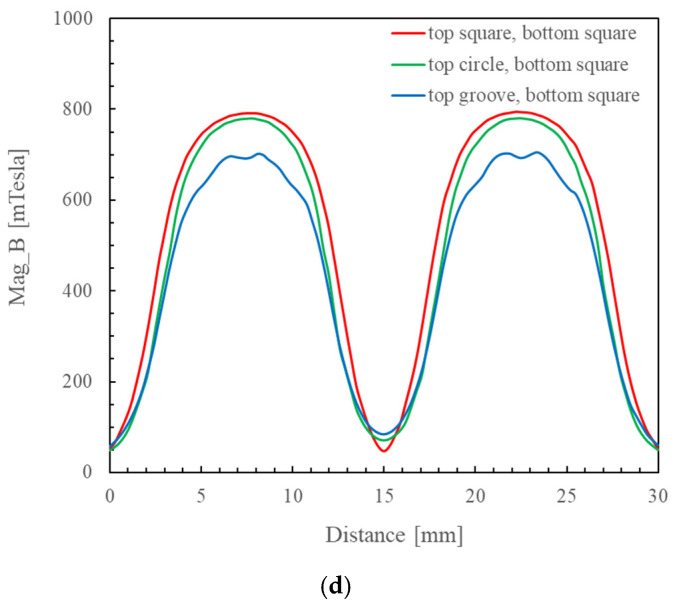
The changes in the continuous magnetic induction intensities at the center cross-section of nine different combinations of dual magnetic poles. (**a**) The magnetic induction intensity between a cubic upper magnetic pole and a cubic lower magnetic pole; (**b**) the magnetic induction intensity between a cylindrical upper magnetic pole and a cubic lower magnetic pole; (**c**) the magnetic induction intensity between a 90°-slotted cylindrical upper magnetic pole and a cubic lower magnetic pole; and (**d**) comparison of the maximum values in the above three situations.

Figure 11a–c show the changes in the continuous magnetic induction intensities at the center cross-section of the nine different combinations of dual magnetic poles. Based on the analysis using Ansys Maxwell, the magnetic induction intensities were quantitatively analyzed, and the maximum magnetic induction intensity occurred at the center of the magnetic poles in all of the dual magnetic pole combinations. The simulation results, respectively, indicated that among the three dual magnetic pole combinations using a cubic upper magnetic pole, the maximum continuous magnetic induction intensity occurred when the cubic upper magnetic pole was paired with a lower cubic pole; among the three dual magnetic pole combinations with an upper cylindrical magnetic pole, the maximum continuous magnetic induction intensity occurred when the upper cylindrical magnetic pole was paired with a lower cubic magnetic pole; and among the three dual magnetic pole combinations with a 90°-slotted cylindrical upper magnetic pole, the maximum continuous magnetic induction intensities occurred when the 90°-slotted cylindrical upper magnetic pole was paired with a lower cubic magnetic pole. From the comparison of the maximum values in the above three situations in Figure 11d, it was obvious that the maximum continuous magnetic induction intensity occurred when a cubic upper magnetic pole was paired with a lower cubic pole.

**Figure 12:**
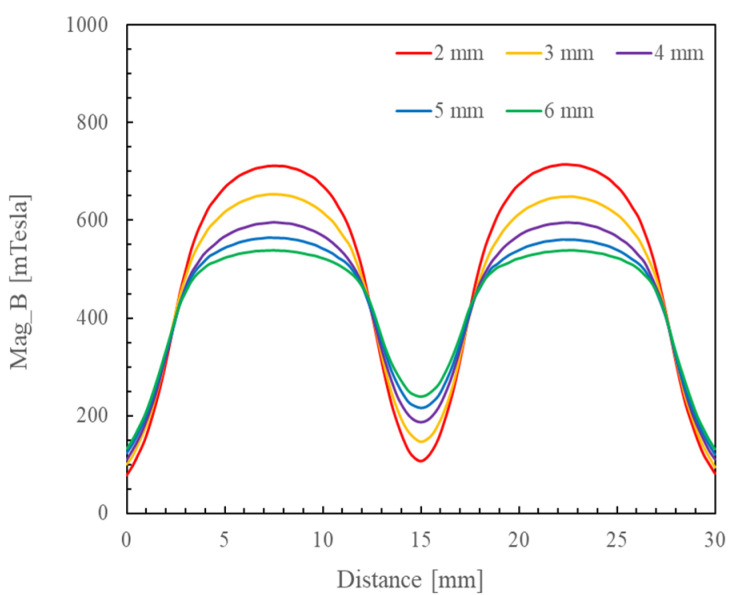
The magnetic induction intensities at the center cross-sections of dual magnetic poles with different working gaps.

Figure 12 shows the magnetic induction intensities at the center cross-sections of the dual magnetic poles with different working gaps. The simulation results indicate the following: The magnetic induction intensity increases as the distance between the upper and lower permanent magnets decreases. Furthermore, the maximum magnetic induction intensity forms at the center of the magnetic pole. According to the classic Formulas (1) and (2) for the magnetic force, the force of the magnetic field is influenced by its magnitude, and the magnetic induction intensity also increases as the magnitude of the magnetic field increases. The polishing pressure of the abrasive particles increases with an increasing force of the magnetic field, which improves the efficiency of polishing the workpiece surface. Therefore, the working gap set between the upper and lower magnetic poles should be shortened as much as practicable to obtain a greater magnetic induction intensity while ensuring normal operation of the upper–lower magnetic poles.

## 4. Discussion

**Figure 13:**
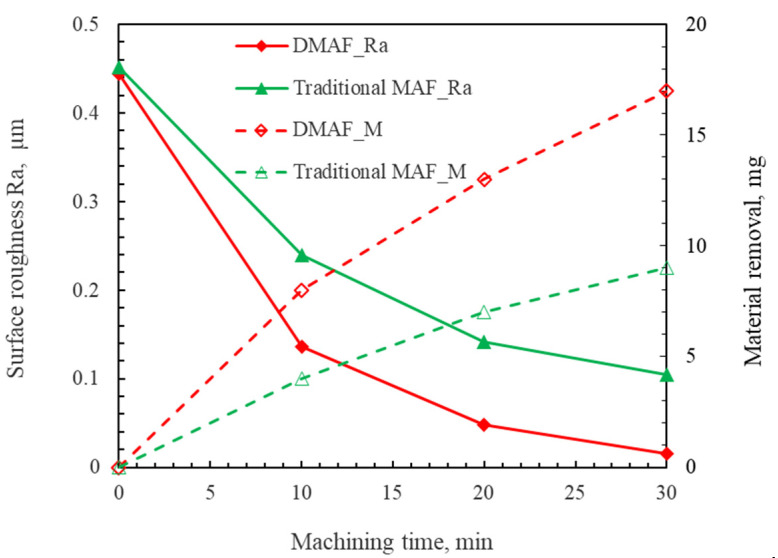
Comparative experimental results for DMAF and traditional MAF.

The comparative experimental results between DMAF and traditional MAF are presented in Figure 13. The findings demonstrate that the material removal efficiency of DMAF is significantly higher than that of traditional MAF, while the surface roughness achieved using DMAF is comparatively lower. These results substantiate that DMAF exhibits a superior polishing efficiency for the TC4 titanium alloy compared to that of traditional MAF.

**Figure 14:**
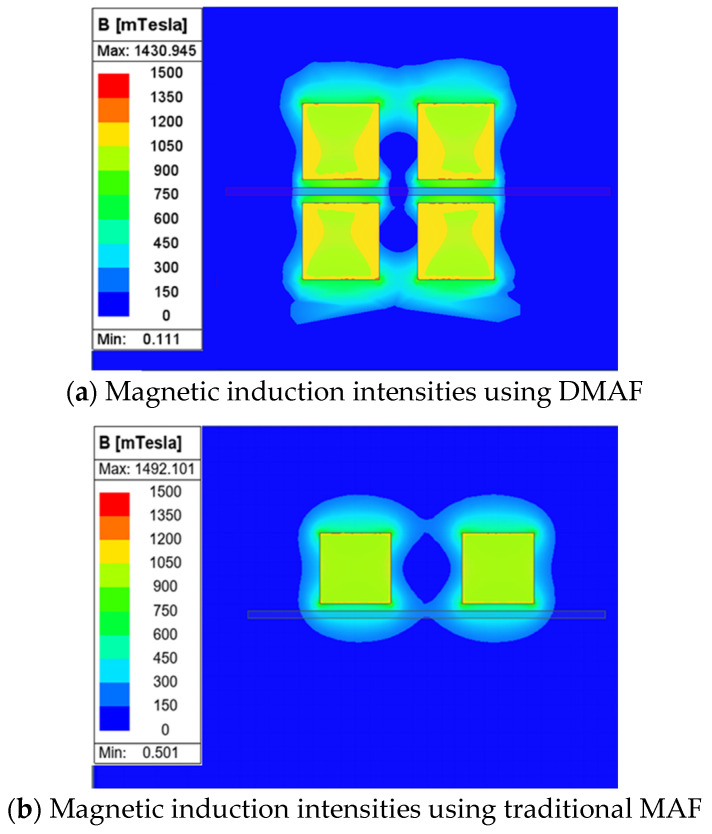
Comparative experimental results of magnetic induction intensities between DMAF and traditional MAF.

The magnetic induction intensities of both methods were simulated using Ansys Maxwell finite element analysis software, with the results of the analysis illustrated in Figure 14. By comparison, it can be seen that the average magnetic induction intensity in the workpiece processing area measures approximately 0.0.44 T with the traditional MAF method, whereas the DMAF method yields a higher value of 0.792 T. The DMAF process exhibits a magnetic induction intensity approximately twice that with the traditional MAF method. According to classical calculation Formulas (1)–(3) for the magnetic force acting on magnetic particles in the magnetic field, it is known that the magnetic field intensity *H* and the magnetic field gradients ∂H/∂x, ∂H/∂y are the main factors affecting the polishing pressure of abrasive particles [29]. *H* is also calculated using Formula (4). The magnetic induction intensity *B* is proportional to that of the magnetic field *H*.

(1) $$F_{x}={KD}^{3}\chi \mu_{0}H\;(\partial H/\partial x)$$

(2) $$F_{y}={KD}^{3}\chi \mu_{0}H\;(\partial H/\partial y)$$

*F*^2^*= F_x_*^2^*+ F_y_*^2^
      (3)

*H = B/µ*
        (4)

where *K* is the correction coefficient, *D* is the diameter of the magnetic abrasive particles, *χ* is the susceptibility of the magnetic abrasive particles, *µ*_0_ is the permeability of a vacuum, *H* is the intensity of the magnetic field, and *∂H/∂x* and *∂H/∂y* are, respectively, the magnetic field gradients in the X direction and the Y direction. *B* is the magnetic induction intensity, and *µ* is the magnetic permeability of the medium.

Therefore, it can be inferred that compared with traditional MAF, the DMAF method significantly enhances the magnetic induction intensity in the processing area. During polishing, the greater the magnetic induction intensity, the greater the force on the magnetic abrasive particles, and the greater the polishing force on the surface of the workpiece. Moreover, the higher the magnetic induction intensity, the stronger the adsorption force of the magnetic abrasive, which is more conducive to the removal of the workpiece material.

## 5. Conclusions

This paper proposes a dual-pole magnetic finishing (DMAF) method for achieving nanoscale polishing of the TC4 titanium alloy’s surface. Through finite element simulation, parameter experiments, and an analysis of the magnetic field, the polishing characteristics and mechanism of the DMAF method have been studied. The following conclusions are drawn:

(1) The distribution of the magnetic field and the magnetic induction intensity generated using different combinations of magnetic poles were analyzed using Ansys Maxwell simulation software. The strongest magnetic induction intensity generated by the upper and lower magnetic poles in the polishing zone, which were both cubic magnetic poles, reached a maximum value of 0.792 T.
(2) By studying the effect of the gap between the upper and lower magnetic poles and the rotational speed of the magnetic poles on the polishing effect, we found that a better surface polishing effect could be obtained using a 5 mm gap between dual-magnetic poles and a 300 rpm rotational speed for the magnetic poles.
(3) Using the same gap between the upper and lower magnetic poles and the same rotational speed of the magnetic poles, when using combination IV of the mixed magnetic abrasive, the surface roughness Ra of the workpiece could be reduced from an initial 0.433 μm to 0.015 μm. Additionally, a mirror-like polishing effect was achieved in the polishing zone on the workpiece surface.
(4) The surface morphology of the workpiece was evaluated using a white light interferometer. The results show that the surface roughness Sa of the measurement point was reduced to below 10 nm, and the 3D morphology of the workpiece’s surface basically tended to become flat. However, a small number of defects still existed on the workpiece surface.

## Figures and Tables

**Table 1:** Experimental conditions.

Machining Parameters	Numerical Value
Workpiece	TC4 titanium alloy plate (100 × 80 × 1 mm)
Initial surface roughness Ra	0.4~0.5 μm
Magnetic pole material	NdFe35 permanent magnet
Mixed magnetic abrasive	Electrolytic iron powder (Fe_3_O_4_): #45 (325 μm), #100 (150 μm), #200 (75 μm), #450 (30 μm);
	WA(Al_2_O_3_): #800 (18 μm), #2000 (6.5 μm), #4000 (3 μm), #8000 (1.6 μm); DMD: #W5 (3 μm), #W2 (1.2 μm), #W1 (0.6 μm);
	Diamond oil-based grinding fluid
Mass ratio of iron-based phase to polishing phase	2:1
Rotational speed of magnetic pole	200 rpm, 250 rpm, 300 rpm
Gap between upper and lower magnetic poles	4 mm, 5 mm, 6 mm
Total polishing time	30 min (each polishing stage is 10 min)

## Data Availability

The original contributions presented in this study are included in the article. Further inquiries can be directed to the corresponding author.
